# Opportunities for resource recovery from Latvian municipal sewage sludge

**DOI:** 10.1016/j.heliyon.2023.e20435

**Published:** 2023-09-26

**Authors:** Ruta Zarina, Linda Mezule

**Affiliations:** a Water Research and Environmental Biotechnology laboratory, Riga Technical University, Kipsalas 6A-263, Riga, Latvia

**Keywords:** Sewage sludge, Resource recovery, Sludge management, Secondary raw materials, Cellulose

## Abstract

Sewage sludge is a type of waste that has high health and environmental risks associated with its reuse. Moreover, sludge has been neglected in global circular economy targets because it is generated in considerably lower quantities than municipal solid waste. At the same time, European Union's transition towards circular economy has set the need to reduce the amount of waste and to promote the production of secondary raw materials. Many countries have developed national strategies for sludge management to reach their sustainability goals. In Latvia, the current sludge management approaches include land application, composting and anaerobic digestion which all utilize sludge as an organic fertilizer. As an alternative to current management practices, resource recovery is put forward as a solution that is in agreement with EU policy. Carbohydrates (including cellulose), proteins and lipids were selected as candidates for energy and materials recovery from sludge. For the first time, this study demonstrates a comprehensive assessment of Latvian municipal sewage sludge composition and offers the theoretical yields of secondary resources on a yearly basis. Primary, secondary, and anaerobically digested sludge from 13 wastewater treatment plants (WWTPs) in Latvia was characterized in this study. The most abundant sludge type – secondary sludge – contained 18.5% proteins, 9.8% lipids and 2.6% cellulose per TS. On a yearly basis, secondary sludge from all Latvian WWTPs could provide 2530 t proteins, corresponding to 750 t protein-based fertilizer. Primary sludge contained 23.9% proteins, 9.1% lipids and 7.1% cellulose per TS. Primary sludge could provide 763 t/a carbohydrates, including 545 t/a cellulose. The currently available secondary and digested sludge would yield 727 t bioethanol, corresponding to 4.0% of the national biofuel consumption. This work applies the concept of resource recovery to the Latvian wastewater sector and shows the potential of simultaneously addressing waste and wastewater management issues.

## Highlights

• First fingerprint of cellulose and macromolecule content in Latvian sewage sludge
• 18.5% proteins, 9.8% lipids and 2.6% cellulose per TS in secondary sludge
• Primary sludge could provide 763 t/a carbohydrates, including 545 t/a cellulose
• Sludge could yield 727 t bioethanol, equal to 4.0% of its consumption in Latvia
• Resource recovery from sludge is a promising strategy for economic independence

## Graphical abstract

**Figure:**
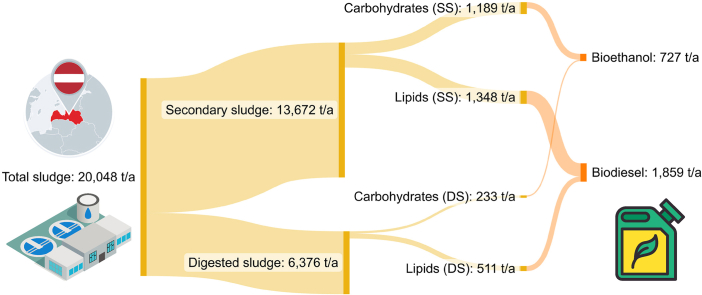

## 1 Introduction

European Union (EU) Green Deal and the United Nations Sustainable Development Goals have set ambitious targets for global reduction in greenhouse gas emissions, increase in energy efficiency, and transitioning towards renewable energy sources. These targets, particularly for waste reduction and recovery, are driving the implementation of resource-efficient practices. An intriguing stream of waste is sewage sludge, a by-product of wastewater treatment with an underestimated potential. In the EU, it is estimated that 7.5 million tonnes of dry sewage sludge were produced in 2021 [1,2]. The annual production of sludge is expected to increase in the future, mainly due to growing urbanization and an increased access to centralized wastewater treatment systems [3]. The majority of sewage sludge in the EU is currently managed by conventional methods – composting, application on agricultural lands, incineration or landfilling [4]. However, the current sludge reuse methods are limited to soil fertilization and heat energy recovery, and they fail to recognize sewage sludge as a valuable resource of recoverable energy and chemical compounds [5]. Additionally, there are concerns over the safety of sludge as a direct soil amender due to the potential presence of pathogens, microplastics, per- and polyfluoroalkyl substances and other contaminants [6,7]. Thus, to avoid the introduction of untreated sludge into the environment, it is ever so important for sludge management practices to be focused on resource recovery. Various novel technologies of sludge resource recovery are available yet they have not been widely implemented at wastewater treatment plants (WWTPs), such as cellulose, heavy metal, polyhydroxyalkanoate and protein recovery [[8], [9], [10]]. A typical European WWTP may currently practice energy recovery (through anaerobic digestion) and, increasingly, phosphorus recovery [11]. In the future, WWTPs are expected to become water resource recovery facilities as part of their efforts to implement circular economy [12].

In 2022, 20 thousand tonnes of dry sewage sludge were produced in Latvia. Nearly half of the nationally produced sludge (46%) was in temporary storage, while the remaining sludge was used as soil fertilizer, including direct application on agricultural land (13%) and composting (5%), and 33% was treated in other ways (including anaerobic digestion) [13]. Sludge utilization methods that were not practiced in 2022 include landfilling, land recultivation and landscaping. Recently, the Latvian water and wastewater works association has developed a national management strategy for sewage sludge as part of the EU's Circular Economy Action Plan [14]. The main goals of this strategy are decreasing the amount of sludge in temporary storage and landfills, and increasing the amount of sludge that is reused according to the principles of circular economy. Due to price competitiveness, composting and soil incorporation were selected as the primary modes of sludge management in Latvia. Similarly as in other EU countries, there is still a lack of novel, high value-added methods of sludge reuse in Latvia. Therefore, sludge valorisation through resource recovery appears to be a promising strategy to improve the economic and environmental performance of Latvian WWTPs.

By recognizing the organic nature of sludge, three main groups of macromolecules, namely, proteins, lipids and carbohydrates, emerge as good candidates for resource recovery [15]. Additionally, cellulose as a specific fraction of carbohydrates is intriguing because of its overlooked presence in sludge and possible biotechnological applications [16]. Up to 40% of influent wastewater suspended solids is attributed to cellulose fibres from toilet paper [17]. If recovered, the material can be used as a feedstock for the production of various chemicals and biofuels. Proteins can become fertilizers [18], animal feed [19] and foaming agents [20]. Lipids can be converted into biodiesel [21] or used for the extraction of valuable chemicals such as ceramide [22]. Fermentation of carbohydrates can yield various biofuels such as bioethanol [23,24], biobutanol [25], biodiesel [26] and biogas [27]. Sewage sludge is often disregarded as a possible feedstock for second generation bioethanol even though its cellulose content is recognized [28]. Alternatively, recovered cellulose can be used for the production of construction materials [29], nanocellulose [30] and chemical building blocks [31]. Considering sewage sludge in broad terms, the organic fraction is useful for its carbon content whereas the inorganic fraction is valuable as a source of phosphorus. Biochar, a carbon-rich material, can be produced through pyrolysis of sludge, and has many valuable applications such as soil fertilization and water decontamination [32]. Phosphorus is an important plant nutrient, and its recovery is a well-established part of sludge management as it enables phosphorus recycling [33].

As an EU member state, Latvia has implemented biological treatment of urban wastewater to a very high extent, concurrently generating sewage sludge that requires environmentally safe treatment. This study was performed to facilitate the development of a novel and evidence-based sludge management policy in Latvia. In this study, a macromolecular fingerprint of Latvian sewage sludge was obtained for the first time. A comprehensive analysis of sewage sludge chemical characteristics was performed, and future opportunities for the recovery of proteins, lipids, carbohydrates and cellulose were proposed. Based on the chemical analysis results, the theoretical yields of sludge-derived biofuels and chemicals such as bioethanol, biodiesel and phosphorus were quantified. The obtained yields were compared to the current national consumption of relevant resources in order to examine the possibility of achieving energy independence.

## 2 Materials and methods

### 2.1 Latvian wastewater treatment system

In the spotlight of this study is Latvia, a country in north-eastern Europe with a population of roughly 1.9 million people. The annual gross domestic product was 20.7 thousand euros per capita in 2022 [34]. 64% of the population lives in cities [35], and 89% of the urban population has access to centralized sewage systems [36]. The urban wastewater generated in Latvia has a population equivalent of 1.5 million, and 99% of it is treated in compliance with the requirements of Urban Wastewater Treatment Directive [37].

Thirteen WWTPs of various sizes were included in the assessment (described in Table 1). The collected sewage sludge samples represent 71% of the wastewater treated and 67% of the sludge produced in Latvia in 2022 [13]. The largest WWTP was responsible for 45% of the wastewater treated and 37% of the sludge produced nation-wide in 2022. Population equivalent (PE) of the 12 smaller WWTPs was between 10 000 and 70 000 whereas for the largest plant PE reached almost 700 000. There are 57 WWTPs in Latvia with a population equivalent higher than 2000 [37], meaning that the sampled locations cover 23% of the Latvian WWTPs considered under Urban Wastewater Treatment Directive. Additionally, there are approximately 900 smaller WWTPs in Latvia that serve communities with less than 2000 PE [13].

**Table 1:** Characteristics of selected Latvian WWTPs [13].

Label	Population equivalent	Annual flow, millions m^3^	Annual sludge production, dry tonnes	Influent COD, mg/L	Share of industrial wastewater	Share of domestic wastewater	Industries
**A**	10780	2.4	885	281	18%	67%	Port services, petroleum product handling, fish
**B**	12370	0.4	90	1089	8%	78%	Grain processing, hospital, household and construction chemicals
**C**	13830	1.2	365	414	47%	50%	Industrial park, chemical and pharmaceutical production
**D**	15450	0.6	254	1222	33%	67%	Military base, snacks, fish, alcoholic beverages
**E**	16140	1.0	104	848	0%	53%	Hospitality
**F**	28690	1.5	454	934	17%	60%	Dairy, beer, metalworking, grain processing
**G**	28770	2.4	394	644	8%	44%	Woodworking, grain processing
**H**	30850	2.4	626	615	5%	59%	Tourism, health resorts, confectionery
**I**	37820	0.9	450	1431	24%	58%	Dairy, confectionery, ice cream
**J**	51240	3.3	909	680	11%	60%	Dairy, meat, heavy machinery components
**K**	55820	5.4	601	565	6%	54%	Textiles, hospital, metalworking, port services, fish
**L**	66170	4.0	1142	831	19%	78%	Dairy, bakery, meat, driving chains, rail car maintenance
**M**	697270	48.9	9644	798	15%	55%	Dairy, landfill, confectionery, fish, beverages, plywood, pharmaceuticals, meat, port services

The share of industrial and domestic wastewater was calculated based on public reports for 2020 or was provided by the WWTP for 2021. Flow inputs from rainwater or infiltration are not included in the table.

A typical WWTP in Latvia performs biological wastewater treatment by activated sludge process. Excess secondary sludge is dewatered by centrifugation and is typically stored in open-air fields before being composted, applied on agricultural lands, or sent to nearby biogas plants. Only one Latvian WWTP (M) operates a primary clarifier prior to the biological treatment, thus being the sole producer of primary sludge in the country. WWTP M also operates an on-site anaerobic digester where a mix of primary and secondary sludge is utilized for biogas production. The final digested sludge from this WWTP is dewatered via centrifugation and sent to open-air sludge fields for further storage or composting.

Municipal WWTPs in Latvia typically receive a small share of industrial wastewater. One of the sampled locations (WWTP E) does not receive any industrial discharges while WWTP C receives 47% industrial wastewater. The most common industries contributing to the sampled WWTPs were dairy, beverages and food production (Table 1).

### 2.2 Sewage sludge samples

Sewage sludge samples were collected from January to March 2022 in 13 municipal WWTPs in Latvia. Locations were selected to cover a wide range of industries contributing to the sewerage network and to represent all regions of Latvia. Vidzeme region (north-central Latvia) was represented by six WWTPs, Zemgale region (south-central) – by three WWTPs, Kurzeme (west) and Latgale (east) regions – each by two WWTPs. WWTPs requested to remain anonymous and thus were assigned labels WWTP A to WWTP M. Five different types of sludge were collected: excess secondary sludge (ESS; n = 12), dewatered secondary sludge (DSS; n = 11), primary sludge (PS; n = 1), digestate (n = 1), anaerobically digested sludge (dewatered digestate; ADS; n = 1). Primary sludge, digestate and digested sludge all originate from WWTP M. An in-depth information on population equivalent, annual wastewater flow, annual sludge production, yearly average influent chemical oxygen demand (COD), share of domestic and industrial wastewater relative to the flow, and the main industries connected to the municipal sewerage network for the sampled WWTPs is provided in Table 1.

All samples were collected in plastic containers, kept on ice during transportation, split in aliquots upon arrival in the lab and stored at −18 °C until analysis.

### 2.3 Analytical methods

#### 2.3.1 Total, fixed and volatile solids

Total solids (TS) were determined by drying the samples in an oven at 105 °C for 12 h. Fixed and volatile solids (FS, VS) were determined in previously dried sludge (at 105 °C) by heating the samples in a muffle furnace (*Nabertherm B180*) at 550 °C for 4 h.

#### 2.3.2 Carbohydrates

DuBois method [38] was used for the determination of carbohydrate content in sludge samples. Approximately 0.1 g dry sludge was used for the analysis. Samples were prepared by adding 3% sulfuric acid and autoclaving at 121 °C for 15 min. Sample extracts were neutralized with sodium hydroxide, diluted with purified water to 50 mL and centrifuged at 8000 rcf for 10 min. The obtained supernatant was analysed by a UV/VIS spectrophotometer at a wavelength of 490 nm. Glucose was used as the reference compound. The detection range was 0.02–0.10 mg glucose equivalents per mL.

#### 2.3.3 Proteins

Protein content was determined by the Lowry method [39]. Reagents were prepared in concentrations described by Shen et al. [40]. Approximately 0.1 g dry sludge was used for the analysis. Samples were treated with a probe sonicator for 2 min (130 W, 20 KHz, 30% amplitude, *Cole-Parmer*, USA), diluted to 20 mL with phosphate buffered saline solution (pH 7.4), heated in a water bath for 15 min, centrifuged at 10000 rcf for 15 min. The supernatant was analysed by a UV/VIS spectrophotometer at a wavelength of 750 nm. Bovine serum albumin (BSA, *Sigma Aldrich*) was used as the reference compound. The detection range was between 0.05 and 0.52 mg BSA equivalents per mL.

#### 2.3.4 Lipids

Lipid content was determined by sulfo-phospho-vanillin method [41]. Approximately 0.02 g dry sludge was used for the analysis. Samples were diluted with 5 mL purified water and treated with a probe sonicator for 1 min (130 W, 20 KHz, 30% amplitude). Then, 5 mL methanol and 5 mL chloroform were added to the sample solution. The samples were mixed by vortexing and shaking intensively three times in 10 min intervals, followed by centrifugation at 8000 rcf for 10 min. The lower layer of chloroform extract was collected separately. Between 0.1 and 0.4 mL sample extract was then heated to evaporate the solvent. 0.1 mL concentrated sulfuric acid was added and the heating was continued until a brown coloration appeared. After adding 2.4 mL phosphovanillin reagent, the sample was incubated for 10 min at room temperature and then analysed by a UV/VIS spectrophotometer at a wavelength of 530 nm. Olive oil (*Acros Organics*) was used as the reference compound. The detection range was 11–87 μg lipids.

#### 2.3.5 Cellulose

Cellulose content was determined by the Schweizer method following the protocol by Gupta et al. [42] with some modifications for scaling down to 50 mL centrifuge tubes. For approximately 0.1–0.3 g dry sludge samples, 10 mL Schweizer's reagent (*VWR Chemicals*) was used to dissolve the cellulose. Approximately 30 mL 80% ethanol was used to precipitate the cellulose. The obtained cellulose precipitate was washed with dilute hydrochloric acid several times and then transferred onto pre-dried and pre-weighed glass fibre filters (47 mm diameter, *Merck Millipore*). The filters were dried overnight at 105 °C, then heated at 500 °C for 2 h. Cellulose content was calculated as the difference between the filter mass before and after heating at 500 °C, and expressed as mg cellulose per mg TS.

#### 2.3.6 Statistics

TS, protein, lipid and carbohydrate analyses were performed in triplicate for each sludge sample. One or two repeats per sample were performed for FS and cellulose analysis and therefore are only presented as sludge group averages. Results are presented as the mean value ± standard deviation for each sludge type group. Comparisons and correlations between groups were assessed by a *t*-test and Pearson's correlation coefficient.

### 2.4 Quantification of recoverable resources

The amount of Latvian sewage sludge used for estimating the resource yields was 7665 t primary sludge, 13672 t secondary sludge, and 6376 t digested sludge. The values were calculated based on the data from the national environmental database 2-Water for year 2022 and the data provided by WWTP M for year 2022 [13]. The amount of resources that could be recovered from Latvian municipal sewage sludge was calculated based on the chemical characterization results described in sections 3, 3.2.3 (Table 2). The annual yield of sludge-derived secondary resources was calculated based on experimental reports or theoretical conversions (Table 3), and is shown in Table 4. “Other organics” was calculated by subtracting the carbohydrate, protein, lipid and ash content from the total solids. Cellulose was assumed to be a part of the carbohydrate fraction. All non-cellulose carbohydrates were assumed to be glucose. Lipids were assumed to be palmitic acid triglycerides. Bioethanol and biodiesel yield estimates were based on theoretical reaction equations. The conversion of sludge resources to nanocellulose, phosphorus, biochar and amino acid chelated trace element fertilizer (AACTEF) was based on experimental reports.

**Table 2:** The amount of recoverable resources in Latvian municipal sewage sludge.

Total amount	Primary sludge		Secondary sludge		Digested sludge	
	Content, %	t/a	Content, %	t/a	Content, %	t/a
		7665		13672		6376
Organics	77.4	5935	78.6	10740	66.1	4214
Carbohydrates	10.0	763	8.7	1189	3.7	233
Cellulose	7.1	545	2.6	353	1.1	73
Non-cellulose	2.8	218	6.1	836	2.5	160
Proteins	23.9	1831	18.5	2530	8.6	546
Lipids	9.1	701	9.8	1341	8.0	509
Other organics	34.4	2640	41.5	5680	45.9	2926
Inorganics	22.6	1730	21.4	2932	33.9	2162

All values in dry weight, data for year 2022. Primary and digested sludge originates from WWTP M. Secondary sludge refers to sludge from all Latvian WWTPs.

**Table 3:** Mass conversion coefficients for estimating the yield of sludge-derived secondary resources.

Resource → Product	Mass conversion coefficient	Notes
**Carbohydrates → Bioethanol** **Non-cellulose → Bioethanol**	0.51	1 mol glucose produces 2 mol ethanol in a fermentation reaction
**Cellulose → Nanocellulose**	0.334	Cellulose from wastewater solids was converted to nanocellulose as described by [30]
**Proteins → Amino acid chelated trace element fertilizer (AACTEF)**	0.30	Fertilizer production from sludge proteins as described by [18]
**Lipids → Biodiesel**	1.01	1 mol palmitic acid triglyceride produces 3 mol palmitic acid methyl ester in a transesterification reaction
**Ash → Phosphorus**	0.089	Phosphorus content in municipal sewage sludge ash [66]
**Organics → Biochar**	0.85 (primary sludge) 0.77 (secondary sludge) 0.87 (digested sludge)	Biochar yields reported by [67]

**Table 4:** The amount of secondary resources in Latvian municipal sewage sludge.

	Primary sludge	Secondary sludge	Digested sludge	Sum of secondary and digested sludge
Resource → Product	t/a			
Carbohydrates → Bioethanol	390	608	119	727
Cellulose → Nanocellulose	182	118	24	143
Non-cellulose → Bioethanol	112	427	82	509
Proteins → AACTEF	543	750	162	912
Lipids → Biodiesel	704	1348	511	1859
Ash → Phosphorus	154	261	192	453
Organics → Biochar	5045	8270	3666	11936
Other organics → Biochar	2244	4374	2546	6919

All values in dry weight. Conversions are based on the amounts in Table 2 and the coefficients listed in Table 3. Primary and digested sludge originates from WWTP M. Secondary sludge refers to sludge from all Latvian WWTPs. AACTEF – amino acid chelated trace element fertilizer.

## 3 Results & discussion

### 3.1 Proximate analysis

**Fig. 1:**
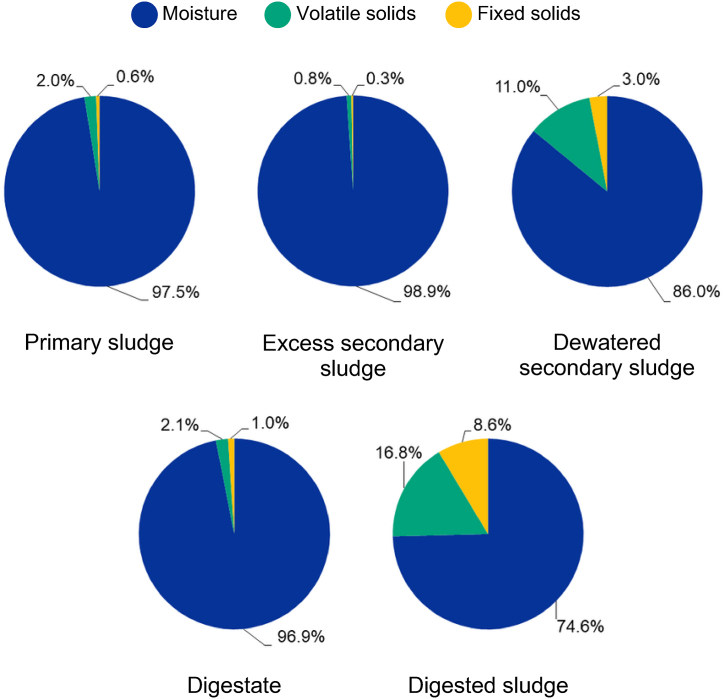
Proximate analysis of Latvian municipal sewage sludge (as percent of wet weight).

The distribution of moisture and solids in different sludge types is shown in Fig. 1. As expected, dewatered sludge types contained the highest levels of total solids: 14.0 ± 0.6% in dewatered secondary sludge and 25.4 ± 1.0% in anaerobically digested sludge. TS content in non-dewatered sludge types was lower: 2.5 ± 0.5% for primary sludge, 3.1 ± 0.1% for digestate, and 1.1 ± 0.2% for excess secondary sludge.

Volatile solids content was slightly higher for the sludge samples collected prior to anaerobic digestion. VS content in PS was 77.4 ± 1.0% TS, while in ESS it was 72.1 ± 6.1% TS and in DSS it was 78.6 ± 3.3% TS. In digestate and digested sludge, the content of VS was 66.8 ± 0.9% TS and 66.1 ± 0.2% TS, respectively. The decrease in VS reflects the degradation of organic solids during anaerobic digestion [43]. In this case, by taking into account the amount of primary, secondary and digested sludge generated at WWTP M per year, it was calculated that the VS reduction after digestion was 58% by weight.

Dewatering of the secondary sludge led to a slight increase in VS content (72.1–78.6% TS) which could be related to the addition of organic-based flocculants. Latvian WWTPs commonly use cationic polyacrylamide polymers to improve sludge dewaterability. The content of cationic polyacrylamide flocculant in dewatered sludge is usually between 0.25 and 1.0% TS [44]. In this case, VS content in secondary sludge increased by 6.5% after dewatering. Thus, other factors are also at play for the changes in organics content, such as sample collection without considering the hydraulic retention time.

Primary [45], secondary [46] and anaerobically digested sludge [47] has been well characterized. The sludge samples collected in Latvian WWTPs had representative values in terms of the distribution of moisture, organics, and inorganics.

### 3.2 Sludge macromolecular composition

**Fig. 2:**
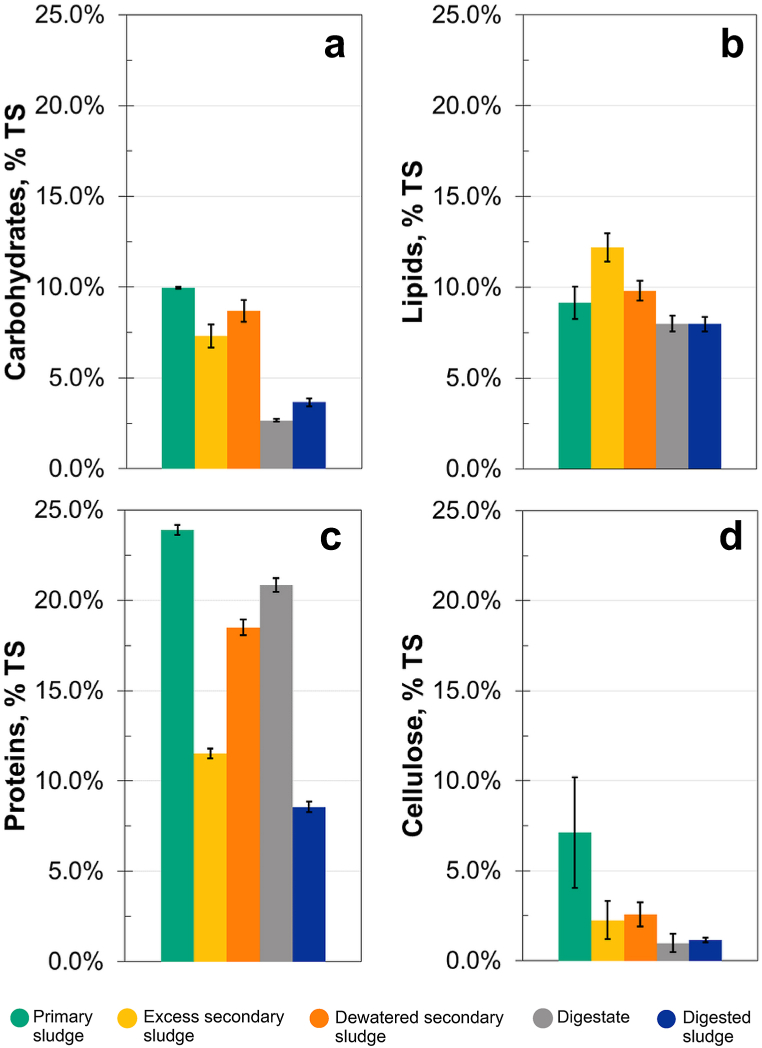
Chemical composition of Latvian municipal sewage sludge (as percent of total solids). a – carbohydrates, b – lipids, c – proteins, d – cellulose.

The content of macromolecules was determined in all five types of collected Latvian municipal sewage sludge (Fig. 2). In general, residual flocculants remaining in the sludge is one of the reasons why excess and dewatered secondary sludge are different in their chemical composition even when expressing the results on a dry basis. Additionally, dewatering by centrifugation may remove some soluble compounds along with the excess moisture. This removal might disproportionally affect some macromolecules but not others.

In this study, digestate and digested sludge samples were created by subjecting a mix of primary and secondary sludge from WWTP M to anaerobic digestion. Approximately half of the feedstock sludge mass was lost after the digestion due to macromolecule degradation and gas formation.

#### 3.2.1 Carbohydrates

According to the results, PS had the highest carbohydrate content (10.0 ± 0.1% TS) (Fig. 2a). This is likely due to paper fibres as well as plant matter from household waste settling in the primary clarifier [17,48]. However, the obtained carbohydrate yield from PS was lower than reported in other studies. For example, PS from a WWTP in Spain contained 26.2%–31.3% TS carbohydrates [21,45].

Secondary sludge contained slightly less carbohydrates than primary sludge. The average content of carbohydrates in ESS from Latvian WWTPs was 7.3 ± 0.6% TS, whereas in DSS it was 8.7 ± 0.6% TS (Fig. 2a). Other studies report similar results, with carbohydrates in secondary sludge generally being around 8–9% TS [49,50].

Carbohydrate levels in the digestate and digested sludge were 2.7 ± 0.1% TS and 3.7 ± 0.2% TS, respectively. The amount of carbohydrates decreased by 81% during anaerobic digestion at WWTP M.

#### 3.2.2 Proteins

Primary sludge had the highest protein content at 23.9 ± 0.3% TS (Fig. 2c). Similarly, primary sludge from a Spanish WWTP has been reported to contain 24.2–27.2% TS protein [21,45]. Protein level in ESS was 11.5 ± 0.5% TS, whereas in DSS it was on average 18.5 ± 0.4% TS. Secondary sludge typically contains more proteins than PS [51], in contrast to our findings. The increased protein content in Latvian PS could be caused by the influent wastewater containing solids from food production.

Digestate contained a moderate amount of proteins, 20.9 ± 0.4% TS, whereas for digested sludge it was only 8.6 ± 0.3% TS. The mass of proteins decreased by 80% during anaerobic digestion at WWTP M.

In nearly all of the sampled WWTPs the protein content of secondary sludge increased after dewatering, whereas for digested sludge the protein content decreased after dewatering. On the one hand, protein content is expected to decrease after sludge centrifugation as this process removes soluble microbial products and a part of loosely-bound extracellular polymeric substances (such as proteins) via the filtrate [52]. On the other hand, dewatering through conditioning and centrifugation does not remove all of the moisture within sludge. In fact, DSS was still composed of 86.0% moisture on average. Additionally, sludge aggregation is led by tightly-bound extracellular polymeric substances which are largely composed of proteins, explaining the disproportional presence of proteins in dewatered sludge [53]. Another consideration is the presence of flocculants in dewatered sludge and their impact on the protein analytical assay. Cationic polyacrylamide polymers are commonly added to secondary sludge in Latvian WWTPs, leading to their presence in dewatered sludge [44]. Lowry assay is based on the interaction between the copper ions and the amide (peptide) bond [54], indicating a feasible mechanism for response overestimation in cases where polyacrylamide flocculants are applied.

#### 3.2.3 Lipids

Lipid content in PS was 9.1 ± 0.9% TS, 12.2 ± 0.8% TS in ESS, and 9.8 ± 0.5% TS in DSS (Fig. 2b). Digestate and digested sludge both contained 8.0 ± 0.4% TS lipids (Fig. 2b). Others report 20–30% TS lipids in PS [21,23,45] and up to 40% TS lipids with optimized extraction conditions [55]. Generally, secondary sludge is reported to contain various levels of lipids, from 4.5% TS [49] to 17.5% TS [23].

The amount of lipids in sludge decreased by 58% during anaerobic digestion. It has been shown experimentally that anaerobic digestion is able to biodegrade nearly 90% of fats, resulting in close to zero percent crude fat in the digestate [56]. The suboptimal reduction indicated that lipid digestion did not occur efficiently at WWTP M. It has been shown that the degradation of lipids starts to occur after 8–10 days of anaerobic digestion treatment [43]. The sludge retention time in the anaerobic digester at WWTP M is apparently shorter than the recommended period. It has been suggested that the main reason for digesting sewage sludge is the benefit of volume reduction and not necessarily the production of biogas [57]. Perhaps for this reason WWTP M has not optimized the anaerobic digestion process parameters and achieved a complete lipid degradation.

### 3.3 Cellulose

The overall pattern in cellulose content (Fig. 2d) was similar to that of carbohydrate content (Fig. 2a). PS had the highest cellulose content at 7.1 ± 3.1% TS. This amount is largely attributed to cellulose particles from toilet paper settling in the primary clarifier [17]. Elsewhere, PS typically contains 18–29% TS cellulose [23,42].

The mean cellulose content in ESS was 2.3 ± 1.1% TS, whereas DSS contained 2.6 ± 0.7% TS cellulose. Typical values for cellulose content are between 0.3% TSS and 9–10% VS in ESS [56,58], and between 1.0% TSS and 13.8% TS for DSS [23,58].

Digestate and digested sludge contained similar levels of cellulose: 1.0 ± 0.5% TS and 1.1 ± 0.1% TS, respectively. In this study, the amount of cellulose in sludge was reduced by 89% during anaerobic digestion. The extent of cellulose biodegradation following anaerobic treatment is around 60% [56].

It was expected that the cellulose content would be higher in the secondary sludge from WWTPs that receive largely domestic wastewater (as opposed to a high industrial wastewater flow) due to toilet paper consumption by residents. However, the correlation between industrial wastewater flow and cellulose content in ESS or DSS was not strong (r = −0.109 and r = −0.311, respectively). The share of domestic wastewater did not strongly correlate to cellulose content either (r = −0.303 and r = −0.151 for ESS and DSS, respectively). The volume of different types of wastewater does not appear to be a good predictor for cellulose-rich sludge. There are several explanations for this. Firstly, industrial wastewater may contain some domestic wastewater, making the difference between the two types of wastewater not as prominent. Additionally, some establishments may encourage disposing of toilet paper in a separate bin, thus reducing the amount of cellulose entering domestic wastewater.

Overall, cellulose content in Latvian sludge was lower than reported in other studies. It has been noted that cellulose content is lower in combined sewerage systems compared to separate systems due to inorganic particle contribution by rainwater [48]. In most cases, the agglomerations in Latvia have a combined sewerage system where the rainwater along with domestic and industrial wastewater is directed to the municipal WWTP. Another reason is the lack of cellulose-rich industrial wastewaters. There is no paper industry in Latvia, whereas the textile industry is present only in a few cities (out of the sampled locations, WWTP K received 4% of its flow from a textiles manufacturer). Furthermore, a lower cellulose content could be related to a lower consumption of toilet paper in Eastern Europe compared to Western European and Northern American countries [16].

### 3.4 Recoverable resources from Latvian sewage sludge

The total amount of secondary sludge generated across all WWTPs in Latvia was 13.7 thousand tonnes in 2022. Additionally, 7.7 thousand tonnes of PS and 5.1 thousand tonnes of DSS were produced at WWTP M and fed to on-site anaerobic digesters, generating 6.4 thousand tonnes of digested sludge. The mass of resources from sludge such as carbohydrates, cellulose, proteins, lipids and ash available in Latvia on a yearly basis is shown in Table 2.

The highest amounts of resources are available in secondary sludge due to its high abundance. The only exception is cellulose which was the most abundant resource in primary sludge (545 t/a), exceeding the yields from secondary or digested sludge (353 and 73 t/a, respectively). Out of the quantified macromolecules, the resource with the highest abundance in Latvian sludge was proteins. PS would supply 1831 t/a proteins, secondary sludge – 2530 t/a, and digested sludge – 546 t/a.

Out of all the sludge types, digested sludge contained the lowest amounts of organic resources (carbohydrates, lipids and proteins) (Table 2). The mediocre yields are partially due to its lower feedstock supply but are also related to the fact that digested sludge has already undergone resource recovery in the form of anaerobic digestion. The valuable organic fraction of sludge has been converted into biogas, and the residual digested sludge is less abundant in macromolecules. To fully exploit the chemical value of digested sludge, the application of a second recovery procedure may take place, such as biochar production or phosphorus recovery. Furthermore, a two-stage sludge treatment process combining anaerobic digestion and bioethanol fermentation has been investigated [59], and was found to be more energy efficient than bioethanol fermentation alone. The advantages of a dual resource recovery approach include a diversification of the biofuels produced and a decrease in waste generation. In the case of Latvia, the majority of WWTPs do not have anaerobic digesters. During their transition to circular and efficient practices, these WWTPs will have to choose whether to implement anaerobic digestion. The combined use of anaerobic digestion and resource recovery may prove to be beneficial for the environmental and economic performance of a WWTP, however, its success depends greatly on the feedstock chemical composition. On the other hand, a recent analysis showed that a WWTP upgrade beyond biogas production is not economically viable under current circumstances [12]. The authors considered nitrogen oxide emission control, biogas upgrading, phosphorus recovery, improved primary clarification and enhanced nitrogen removal, and found that only N_2_O control improved environmental performance while also providing financial benefits. It is clear that balancing the environmental benefits and the financial costs of sludge resource recovery is challenging. Further techno-economic analysis of the resource recovery pathways proposed for Latvian sewage sludge would be beneficial.

Various examples of carbohydrate, protein, lipid, and ash conversion to valuable sludge-derived secondary resources such as biofuels and chemicals were quantified in Table 4. It should be noted that PS is currently an intermediate product in wastewater treatment in Latvia. It is subjected to anaerobic digestion and contributes to the production of digested sludge. If resource recovery was applied to primary sludge, the chemical composition of digested sludge would change as well, complicating the calculations. Therefore, due to the current sludge management scenario, the total amount of resources included in further analysis is the sum of resources derived from secondary and digested sludge (i.e., excluding PS).

Biofuels such as bioethanol and biodiesel have a role in achieving the EU's renewable energy targets. The Renewable Energy Directive demands that the share of renewable energy in the final consumption of energy in the transport sector is at least 14% in each Member State, including Latvia. To achieve this, the Latvian government requires fuel retailers to supply diesel with at least 6.5% biofuel additive and petrol with at least 9.5% bioethanol additive. Local retailers offer petrol with 10% bioethanol and diesel with 7% biodiesel. Based on our calculations, it would be possible to recover 1422 t/a carbohydrates from Latvian secondary and digested sludge which could yield 727 t/a bioethanol. To put this into perspective, in 2021, 18 000 t bioethanol was imported and consumed in Latvia as a transport fuel [60]. Sludge-derived bioethanol could cover 4.0% of the national consumption and introduce a local supply of this biofuel additive. The lipid fraction from secondary and digested sludge would amount to 1850 t/a and could be converted into 1859 t/a biodiesel. Biodiesel production reached 71 000 t in 2021 in Latvia, while the domestic consumption of biodiesel as a transport fuel was 38 000 t [60]. Sludge-derived biodiesel could provide for 4.9% of the national consumption in the transport sector and contribute an additional 2.6% of locally produced biodiesel.

Secondary and digested sludge could supply 426 t/a cellulose, whereas primary sludge as a feedstock could provide up to 545 t/a cellulose. In comparison, 11 618 t of recycled paper products were imported in 2022 [61]. Recovered cellulose could cover 3.7–4.7% of the import needs, depending on the source (secondary and digested sludge or primary sludge, respectively). In addition, the superior cellulose yield shows the advantages of cellulose recovery from primary sludge. Considering the partial loss of cellulose during wastewater treatment, the most efficient location for cellulose recovery is prior to the biological treatment [62]. Furthermore, the recovered cellulose could be converted into 143 t/a nanocellulose. The yield of nanocellulose could reach 182 t/a if primary sludge was utilized for the conversion.

Another resource that is important to the economy is fertilizer. By combining Latvian secondary and digested sludge, it would be possible to recover 3076 t/a proteins which could yield 912 t/a sludge-derived fertilizer (specifically, amino acid chelated trace element fertilizer). The quantity of inorganic compounds present in secondary and digested sludge is 5094 t/a which could provide 453 t/a phosphorus. According to the Latvian Agricultural Census (data for year 2019), 12 678 t phosphorus was applied to agricultural lands, including 11 712 t in the form of inorganic phosphorus fertilizer [63]. The amount of phosphorus embedded in Latvian sludge corresponds to 3.9% of the national consumption of this type of inorganic fertilizer. In 2019, nitrogen inputs to soil via fertilizers reached 84 023 t, including 80 718 t nitrogen via inorganic fertilizer and 3305 t nitrogen via organic fertilizer [64]. Assuming 16% nitrogen content, the protein fraction of Latvian secondary and digested sludge (3076 t/a) theoretically contains 492 t nitrogen which could provide 14.9% of the nitrogen inputs by organic fertilizer.

Biochar was considered in this study as a carbon-rich sludge-derived resource. Latvian sewage sludge contains approximately 60–80% organics (Fig. 1), showing a potential for the production of biochar. Overall, Latvian primary sludge could provide 5935 t/a organics in total. Secondary sludge could yield 10740 t/a Total organics, whereas digested sludge could yield 4214 t/a Total organics. This corresponds to a theoretical production yield of 5045 t/a biochar from primary sludge organics, 8270 t/a from secondary sludge organics, and 3666 t/a from digested sludge organics. The total amount of biochar available from secondary and digested sludge feedstocks would reach 11936 t/a. Considering the total consumption of organic fertilizer in Latvia in 2021 (4571400 t/a), the sludge-derived biochar would provide a negligible share (0.3%) of the demand for fertilizer [65]. However, a more realistic approach to biochar production would involve utilizing the residual organics fraction that remains after carbohydrate, protein and lipid recovery. In this scenario, the Other organics fraction would yield 2244 t/a biochar from primary sludge, 4374 t/a from secondary sludge and 2546 t/a from digested sludge. In total, 6919 t/a biochar could be produced from secondary and digested sludge, corresponding to 0.2% of the organic fertilizer consumption.

In principle, the use of sludge-derived resources as fertilizers is similar to the direct use of sludge as a fertilizer. However, the resource recovery approach of fertilizer production (e.g., phosphorus recovery, sludge proteins to AACTEF, or sludge organics to biochar) would significantly reduce the pollution concerns associated with sludge reuse due to the processing involved. Thermal or chemical treatment during sludge-derived fertilizer production is likely to decrease the pathogen load and decompose the organic contaminants.

## 4 Conclusions

Based on the Latvian municipal sewage sludge characterization and the resource recovery calculations, it was found that secondary sludge from Latvian WWTPs is a good candidate for the recovery of proteins and, to a lesser extent, lipids and carbohydrates. In locations where it is available, primary sludge is a prime supply for cellulose recovery. Digested sludge has already undergone resource recovery through biogas production and thus offers lower yields of secondary resources. Sludge-derived bioethanol and biodiesel could provide a small share of the biofuel additives consumed in Latvia and simultaneously address renewable energy, waste and wastewater management issues. Fertilizer obtained by novel resource recovery approaches could lessen the pollution concerns associated with direct land application of sludge. The potential yield of sludge-derived resources was likely overestimated due to the use of proxy coefficients, showing the need for pilot-scale and large-scale experiments testing the feasibility of various sludge resource recovery approaches. The various routes of resource recovery implementation at Latvian WWTPs should be further evaluated by a life cycle analysis and a techno-economic assessment.

## Data availability statement

Data will be made available on request.

## Additional information

No additional information is available for this paper.

## CRediT authorship contribution statement

**Ruta Zarina:** Conceptualization, Formal analysis, Investigation, Methodology, Visualization, Writing – original draft, Writing – review & editing. **Linda Mezule:** Conceptualization, Funding acquisition, Methodology, Project administration, Resources, Supervision, Writing – original draft, Writing – review & editing.

## Declaration of competing interest

The authors declare that they have no known competing financial interests or personal relationships that could have appeared to influence the work reported in this paper.
